# Safety and Efficacy of Cinacalcet in Children Aged Under 3 Years on Maintenance Dialysis

**DOI:** 10.1016/j.ekir.2024.04.061

**Published:** 2024-05-07

**Authors:** Julie Bernardor, Sacha Flammier, Ilona Zagozdzon, Alexander D. Lalayiannis, Linda Koster-Kamphuis, Enrico Verrina, Eiske Dorresteijn, Isabella Guzzo, Dieter Haffner, Rukshana Shroff, Claus P. Schmitt, Justine Bacchetta

**Affiliations:** 1 Department of Pediatric Nephrology, Rheumatology and Dermatology, Reference Center for Rare Renal Diseases, Reference Center for Rare Diseases of Phosphate and Calcium, Femme Mère Enfant Hospital, Hospices Civils de Lyon, Lyon, France; 2 INSERM 1033 Research Unit, Université Claude Bernard Lyon 1, Lyon, France; 3 Department of Pediatric Nephrology, CHU de Nice, Hôpital Archet, Nice, France; 4 Centre for Pediatric and Adolescent Medicine, University Hospital Heidelberg, Im Neuenheimer Feld, Heidelberg, Germany; 5 Department of Pediatrics, Nephrology and Hypertension, Medical University of Gdańsk, Poland; 6 Paediatric Nephrology, Birmingham Women’s and Children’s NHS Foundation Trust, Birmingham, UK; 7 Department of Pediatric Nephrology, University Medical Center, St. Radboud/Radboud University, Nijmegen, The Netherlands; 8 Division of Nephrology, Dialysis, and Transplantation, IRCCS Istituto Giannina Gaslini, Genova, Italy; 9 Department of Pediatric Nephrology, Sophia Children’s Hospital, Erasmus Medical Center, Rotterdam, The Netherlands; 10 Division of Nephrology, Dialysis and Transplantation, Bambino Gesù Children’s Hospital, IRCCS, Rome, Italy; 11 Department of Pediatric Kidney, Liver and Metabolic Diseases, Pediatric Research Center, Hannover Medical School, Hannover, Germany; 12 Pediatric Nephrology Unit, University College London Great Ormond Street Hospital for Children and Institute of Child Health, London, UK

**Keywords:** cinacalcet, infants, kidney failure, secondary hyperparathyroidism

## Abstract

**Introduction:**

Secondary hyperparathyroidism (sHPT) is particularly severe in rapidly growing infants in dialysis. Although cinacalcet is effective and licensed in dialysis in children aged >3 years, its efficacy and safety for children aged <3 years is unknown.

**Methods:**

We identified 26 children aged <3 years who were on dialysis and treated with cinacalcet between 2009 and 2021 in 8 European pediatric centers.

**Results:**

Median (interquartile range) age at the start of cinacalcet was 18 (interquartile range: 11–27) months, serum parathyroid hormone (PTH) was 792 (411–1397) pg/ml, corresponding to 11.6 (5.9–19.8) times the upper limit of normal (ULN). Serum calcium was 2.56 (2.43–2.75) mmol/l, and serum phosphate 1.47 (1.16–1.71) mmol/l. Serum 25-OH vitamin D (25–OHD) was 70 (60–89) nmol/l, 3 children were vitamin D deficient (<50 nmol/l). The initial cinacalcet dose was 0.4 (0.2–0.8) mg/kg/d and the maximum dose was 1.1 (0.6–1.2) mg/kg/d. The median follow-up under cinacalcet was 1.2 (0.7–2.0) years. PTH decreased to 4.3 (2.2–7.8) times the ULN after 6 months, to 2.0 (1.0–5.3) times ULN after 12 months, and to 1.6 (0.5–3.4) times thereafter (*P* = 0.017/0.003/<0.0001, log-transformed PTH). Seven of the 26 infants developed 10 hypocalcemic episodes <2.10 mmol/l. Oral calcium intake was 84% (66%–117%) of recommended nutrient intake at start, 100% (64%–142%) at 3 months and declined to 78% (65%–102%) at 12 months of therapy. Three children developed clinical signs of precocious puberty.

**Conclusion:**

Cinacalcet efficiently controlled severe sHPT in children aged <3 years and was associated with hypocalcemic episodes (similar to what is observed in older children) and precious puberty, thereby mandating meticulous control of calcium (considering nutrition, supplementation, and dialysate) and endocrine changes.

## Graphical abstract

**Figure:**
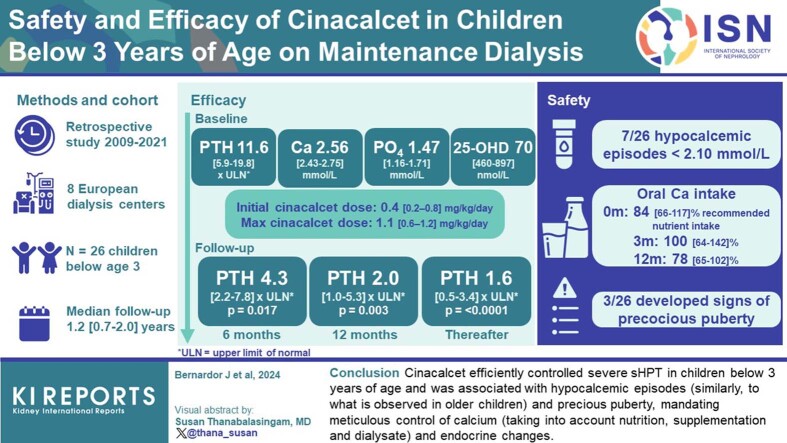

Chronic kidney disease (CKD) is almost inevitably associated with mineral and bone disorder (CKD-MBD), which is characterized by a combination of bone remodeling abnormalities and cardiovascular disease.^1^ The combination of hyperphosphatemia, deficiency in active and/or native vitamin D, and subsequent hypocalcemia induces sHPT, resulting in progressive bone disease and parathyroid hyperplasia.^2^ Several consensus papers and guidelines were published to steer diagnosis and management of pediatric CKD-MBD.^2^, ^3^, ^4^, ^5^ Standard-of-care includes dietary measures, calcium supplementation, phosphate binders, vitamin D sterols, optimization of dialysate calcium, and calcimimetics in children aged >3 years. Evidence is scarce in the youngest children, as reviewed in the recent European consensus on CKD-MBD in infants.^6^ This lack of knowledge is particularly concerning, because infancy is the period of fastest growth, with doubling of body length until the age of 3 years in healthy children and substantial improved growth in CKD children receiving growth hormone therapy from the age of 6 months onward.^7^ Growth-associated highly dynamic bone development explains challenges in CKD-MBD management in this peculiar age group. High nutritional calcium supply is crucial,^8^ but often difficult to achieve in clinical practice, especially in children on a phosphate restricted diet.^9^, ^10^, ^11^ Even with optimized treatment, insufficient MBD control is prevalent, with significant obstacle to bone strength and cardiovascular disease worsening.^12^, ^13^, ^14^, ^15^, ^16^, ^17^ Conversely, the number of small children requiring dialysis is increasing globally,^13^^,^^18^ because kidney replacement therapy has been widely accepted in otherwise stable children from the first days of life onwards.

The calcimimetic cinacalcet potently inhibits PTH synthesis and secretion through the sensitization of the calcium-sensing receptor,^19^^,^^20^ decreasing calcium and phosphate levels, thus potentially reducing the need for parathyroidectomy,^21^ decreasing fracture rates,^22^ and possibly reducing cardiovascular disease in adults on dialysis.^23^^,^^24^ In 2017, Kidney Disease: Improving Global Outcomes recommended calcimimetics as first-line therapy or in combination with vitamin D analogs in dialysis adults.^25^ Two open-label phase 1 studies, 1 open-label phase 2 study, 2 phase 3 randomized studies, and 1 phase 3 single arm extension study have been conducted in a total of 103 pediatric subjects. Seven percent to 57% of the pediatric subjects who received cinacalcet attained PTH levels within recommended target ranges, and 22% to 71% attained a ≥30% reduction in PTH.^26^ Significant reduction of PTH levels was also reported in observational studies in 92 children.^27^, ^28^, ^29^, ^30^, ^31^, ^32^, ^33^ Thus, cinacalcet was licensed in children aged >3 years in Europe; the 2020 European consensus statement suggested routine cinacalcet use in pediatric patients on dialysis who are aged >3 years to treat persistent and severe sHPT, despite optimized conventional management.^5^ However, this treatment remains unlicensed in the USA.

The role of cinacalcet treatment in younger children, with the most rapid bone turnover and mineralization, and consequently the most challenging MBD control, remains nevertheless uncertain. Our aim is to report the European experience of off-label use of cinacalcet in children on dialysis who are aged <3 years.

## Methods

### Patients

We performed a retrospective analysis on cinacalcet use in children on maintenance dialysis, who are aged <3 years, at 35 pediatric dialysis centers between 2009 and 2021; members of the European Society of Pediatric Nephrology and/or the European Rare Kidney Disease Reference Network. Patients were identified and analyzed retrospectively at cinacalcet initiation and every 3 months thereafter for 1 year (when most children reached the age of 3 years) and at last follow-up in 16 patients treated with cinacalcet for more than 1 year. Oral calcium intake from diet and medication was expressed relative to the reference nutrient intake (%RNI) for age,^4^ based on the patients’ actual dry body weight. It was retrospectively reviewed by physicians using the medical charts and the regular nutritional evaluations performed in daily practice. Net dialytic calcium uptake during peritoneal dialysis was estimated from the total volume of dialysis fluid applied and the delta between dialysate calcium concentration and serum calcium concentration (which in average was 0.5 [0.4–0.7] mmol/l), and assuming an average cation equilibration of 55% cation equilibration after 1 hour of dwell time and 80% after 4 hours, as previously demonstrated in children in an randomized controlled trial.^34^ Based upon these uptake data, ultrafiltration-associated calcium losses were estimated to amount to 1.1 mmol/l, with a median dwell time of 75 (interquartile range: 65–90) minutes. Five patients were on hemodialysis at 8 time points. Due to missing data, dialytic calcium exchange on hemodialysis could not be estimated, and these time points were excluded from calcium supply studies. Precocious puberty was defined as the onset of puberty with physical changes, such as breast and pubic hair development.

### Laboratory Analyses

As part of routine follow-up, total calcium, phosphate, total alkaline phosphatase (ALP), PTH and 25-OHD were regularly assessed locally by standard methods. Because of the physiological evolution of plasma phosphate and ALP during childhood, these were expressed as SD score (SDS) for age.^35^^,^^36^ Laboratory parameters were collected at 7 different time points: initiation of cinacalcet therapy (baseline), and at 1, 3, 6, 9, and 12 months, and at last follow-up beyond 12 months in 16 patients. Normal ranges of PTH assays were reported by each center, thus allowing to standardize PTH data relative to the ULN. Our target for PTH levels was set at <3 times the ULN, aligning with current European standards and guidelines.^3^^,^^5^^,^^6^^,^^37^ This threshold reflects a consensus on optimal PTH levels for the management of CKD-MBD in pediatric patients, while recognizing that targets may vary from region to region and according to the decision of the physician. SDS of body weight and SDS body length were assessed at cinacalcet initiation, at 12 months, and at the last follow-up using World Health Organization growth standards.^38^ Vitamin D deficiency was defined as serum 25-OHD less than 50 nmol/l and insufficiency as serum 25-OHD less than 75 nmol/l.^2^

### Ethics

The study was approved by an ethical committee (Comité d’Ethique des Hospices Civils de Lyon, session October 14, 2021, approval 21_634), and declared to the Information Technology and Liberty Commission (CNIL n°21_5634). The study respected European relevant regulations. Three children aged <3 years had been included in an industry-sponsored trial before: data were not published. Approval was obtained from Amgen to include the patients’ local results in this study.

### Statistical Analysis

PTH and PTH-ULN had a non-Gaussian distribution and were log-transformed. Linear mixed model followed by Dunnett’s multiple paired comparisons tests compared to baseline were used for biochemical data. Nonparametric Mann-Whitney tests were used to identify risk factors and to compare patients with hypocalcemic episodes to other patients. Results were described as median (interquartile range), *P*-values <0.05 were considered statistically significant. Kaplan-Meier survival analysis was used to investigate the time-to-attainment of PTH levels 3 times the ULN. GraphPad Prism software 8.0 (GraphPad, La Jolla, CA) was used.

## Results

Eight out of 35 pediatric dialysis centers who participated in the survey reported treating children aged <3 years with cinacalcet between 2009 and 2021. Of 729 children on dialysis in these centers, 194 (27%) were aged <3 years; among them, 26 (13%) received cinacalcet. These infants were followed-up with in Heidelberg (*n* = 14), Gdansk (*n* = 3), Lyon (*n* = 2), Birmingham (*n* = 2), Nijmegen (*n* = 2), Genoa (*n* = 1), Rotterdam (*n* = 1), and Rome (*n* = 1).

### Patients

Demographic, clinical, and biochemical features at cinacalcet initiation are summarized in Table 1. At 18 (11–27) months, 25 children on peritoneal dialysis and 1 on hemodiafiltration (65% of male) started cinacalcet for sHPT, 8 (31%) also displaying hypercalcemia; 23 received enteral tube feeding. Daily calcium intake from diet and medication was 43 (33–63) mg/kg/d, providing 84% (66%–117%) of RNI; dietary phosphate intake was 230 (183–256) mg/d, that is, 47% (40%–52%) of RNI for age. Follow-up period was 1.2 (0.7–2.0) years. Estimated dialytic calcium uptake was 2.4 (1.7–3.1) mg/kg/d. Nine patients received only calcium-based binders (calcium gluconate or calcium carbonate), 2 patients received both calcium-based and calcium-free phosphate binders, whereas 7 patients received only calcium-free binders. All children received vitamin D analogs.

**Table 1:** Individual patient characteristics, nutrition, medication, baseline serum PTH and cinacalcet dose, and follow up times

Patient	Cause of CKD	Age at start of cina (mo)	Dialysis mode	Body weight (kg)	Intake (mg/d, % RNI)		Ca-based binder	Non Ca-based binder	Active vitamin D (μg/d)	PTH (pg/ml)	PTH, (times above ULN)	Ca_c_ (mmol/l)	*P* (mmol/l, SDS)	25-D (nmol/l)	Cina Initial dose (mg/kg/d)	Cina max. dose (mg/kg/d)	f/u (yr), cause of discontinuation
					Ca	P											
1	CNS	7	PD	5.8	290, 100	155, 37	Ca suppl	No	alfacalcidol 1	810	11.3	2.57	1.01, −4.0	69	0.5	1.6	2.4, on-going
2	CNS	8	PD	7.6	352, 92	209, 50	Ca suppl	No	alfacalcidol 1	1245	17.4	2.36	1.55, −1.3	50	0.8	1.0	2.4, KTx
3	CAKUT	9	PD	7.8	693, 178	261, 62	Ca suppl	No	alfacalcidol 0.6	631	8.8	2.21	1.17, −3.2		0.5	0.7	1.0, KTx
4	CAKUT	11	PD	10.4	396, 76		No	No	alfacalcidol 1	563	7.9	2.56	0.95, −4.3		0.3	1.1	1.5, KTx
5	CAKUT	11	PD	6.2	355, 114	167, 40	Ca suppl	No	alfacalcidol 1	852	19.6	2.36	1.63, −0.9	79	0.8	1.2	2.2, KTx
6	Nephronophtis	11	PD	8.6	306, 71	144, 34	Ca suppl	No	alfacalcidol 0.5	410	5.7	2.59	1.13, −3.4	60	0.3	0.4	1.2, KTx
7	CAKUT	12	PD	7.5	3653, 975	254, 51	CaCO_3_	sevelamer	alfacalcidol 1.5	957	14.7	2.55	1.72, −0.5	34	0.1	0.9	2.6, PTH normalization
8	Ischemia	13	PD	8.1	569, 145	233, 47	No	No	alfacalcidol 0.6	773	10.8	2.26	1.23, −2.9		1.2	1.2	0.1, KTx
9	Glycogenosis	14	PD	8.5	533, 132	344, 69	No	No	alfacalcidol 0.2	370	5.2	2.65	1.55, −1.3	65	1.2	1.2	1.7, hypoCa
10	CAKUT	15	PD	12.0		173, 35	No	sevelamer	alfacalcidol 0.8	235	3.5	2.51	1.13, −3.4	97	0.3	0.6	2.5, KTx
11	CAKUT	17	PD	9.0	329, 73	235, 47	No	sevelamer	calcitriol 0.75	525	18.1	2.95	2.18, 1.9	95	0.6	0.6	2.0, KTx
12	CAKUT	18	PD	11.2	422, 75		No	sevelamer	alfacalcidol 1.2	415	6.4	2.43	1.23, −2.9		0.8	0.8	1.6, KTx
13	C- HUS	18	PD	9.5	600, 126		No	No	alfacalcidol 0.4	200	2.8	2.53	1.28, −2.7	72	1.1	1.1	1.8, not efficient
14	ARPKD	8	PD	5.5	267, 91	235, 47	Ca suppl	No	calcitriol 0.5	585	8.2	2.32	1.63, −0.9	68	0.4	1.1	0.9, on-going
15	ADPKD	20	PD	11.4	906, 159	246, 49	Ca suppl	No	alfacalcidol 1.5	170	2.3	2.87	1.66, −0.8	133	0.2	0.3	0.3, on-going
16	ARPKD	28	HDF	10.3	3525, 684	444, 89	CaCO_3_	sevelamer	alfacalcidol 1.2	1164	25.6	2.49	2.01, 1.0	80	0.2	0.2	0.1, hypoCa
17	CAKUT	21	PD	8.5	209, 49	107, 21	No	No	calcitriol 1.5	2320	37.4	2.75	1.16, −3.7	75	0.3	2.1	3.6, KTx
18	CAKUT	23	PD	10.9			No	No	calcitriol 0.25	1397	21.5	2.60	1.16, −3.2	129	0.2	0.2	0.3, PTH normalization
19	ARPKD	25	PD	8.6	176, 41	112, 22	No	sevelamer	alfacalcidol 0.2	236	3.3	3.38	1.38, −2.0	50	0.9	4.0	0.9, hypoCa
20	ARPKD	27	PD	10.5	374, 71	232, 46	No	sevelamer	alfacalcidol 0.5	1829	29.5	2.88	2.81, 5.4	35	0.2	1.7	0.6, KTx
21	Pierson syndrome	28	PD	12.6	623, 99	311, 62	No	No	alfacalcidol 0.5	852	11.9	2.35	1.61, −0.9	65	1.2	1.2	1.3, NA
22	CNS	28	PD	11.1	590, 106	228, 46	Ca suppl	No	alfacalcidol 1	1395	19.5	2.09	1.33, −2.2	70	1.4	1.3	1.9, KTx
23	CAKUT	29	PD	10.9	323, 59	235, 47	No	No	alfacalcidol 0.75	945	32.6	2.75	2.25, 2.5	110	0.3	0.6	0.6, on-going
24	ARPKD	31	PD	13.0	397, 59	297, 59	No	No	calcitriol 2.4	91	1.5	2.77	1.16, −3.1	89	0.1	0.3	0.9, KTx
25	BBS	32	PD	9.1	181, 40	182, 36	No	sevelamer	alfacalcidol 0.2	1422	19.9	2.47	1.95, 0.9		1.1	1.6	0.2, NA
26	CAKUT	35	PD	14.4	547, 76	270, 54	Ca suppl	sevelamer	alfacalcidol 2.5	1757	28.3	2.80	2.45, 3.5	38	0.2	1.1	1.2, on-going

ADPKD, autosomal dominant polycystic kidney disease; ARPKD, autosomal recessive polycystic kidney disease; BBS, Bardet-Biedel syndrome; Ca_c_, albumin corrected calcium; CaCO_3_, calcium carbonate; CAKUT, congenital abnormalities of kidney and urinary tract; C-HUS, congenital hemolytic uremic syndrome; Cina, cinacalcet; CKD, chronic kidney disease; CNS, congenital nephrotic syndrome; f/u, follow-up; HDF, hemodiafiltration; hypoCa: hypocalcemia; KTx, kidney transplantation; NA, not available; N^o^, number; P, phosphate; PD, peritoneal dialysis; PTH, parathyroid hormone; RNI, reference nutrient intake; SDS, SD Score for age; sHPT, secondary hyperparathyroidism; Suppl, supplementation; ULN, upper limit of normal.

Calcium intake from diet and medication fluid was expressed relative to the Reference Nutrient Intake (% RNI) for age.^8^ Between 6 months and 3 years, and between 4 and 5 years, recommended calcium intake are 50 and 44 mg/kg/day, respectively.^8^ Phosphate recommended intakes are 420 and 500 mg, respectively, between 6 and 12 months, and between 1 and 3 years.^4^

### Cinacalcet Dose

In all centers, a suspension provided in capsules was prepared by local pharmacists. The initial cinacalcet dose was 0.4 (0.2–0.8) mg/kg/d, that is, 2 times above the recommended starting dose.^5^ Maximal cinacalcet and last follow-up doses were 1.1 (0.6–1.2) and 1.0 (0.4–1.2) mg/kg/d, respectively. Following cinacalcet initiation, intervals for the first biochemical control varied substantially between centers, ranging from 2 to 3 days (*n* = 14), 7 days (*n* = 7), 10 days (*n* = 1), 2 weeks (*n* = 1) to 1 month (*n* = 1).

### Evolution of CKD-MBD Biomarkers

At cinacalcet initiation, PTH was 792 (411–1397) pg/ml, corresponding to 11.6 (5.9–19.8) times the ULN. Serum calcium concentration was 2.56 (2.43–2.75) mmol/l; 8 patients had hypercalcemia and 3 had hypocalcemia for age.^39^ Serum phosphate was 1.47 (1.16–1.71) mmol/l, with a SDS for age of −1.7 (−3.2 to −0.6]. Total serum ALP and ALP-SDS were 660 (492–905) IU/l and 0.3 (−0.5 to 2.0), respectively; 25-OHD was 70 (60–89) nmol/l, 3 children were vitamin D deficient and 12 were vitamin D insufficient.

**Figure 1:**
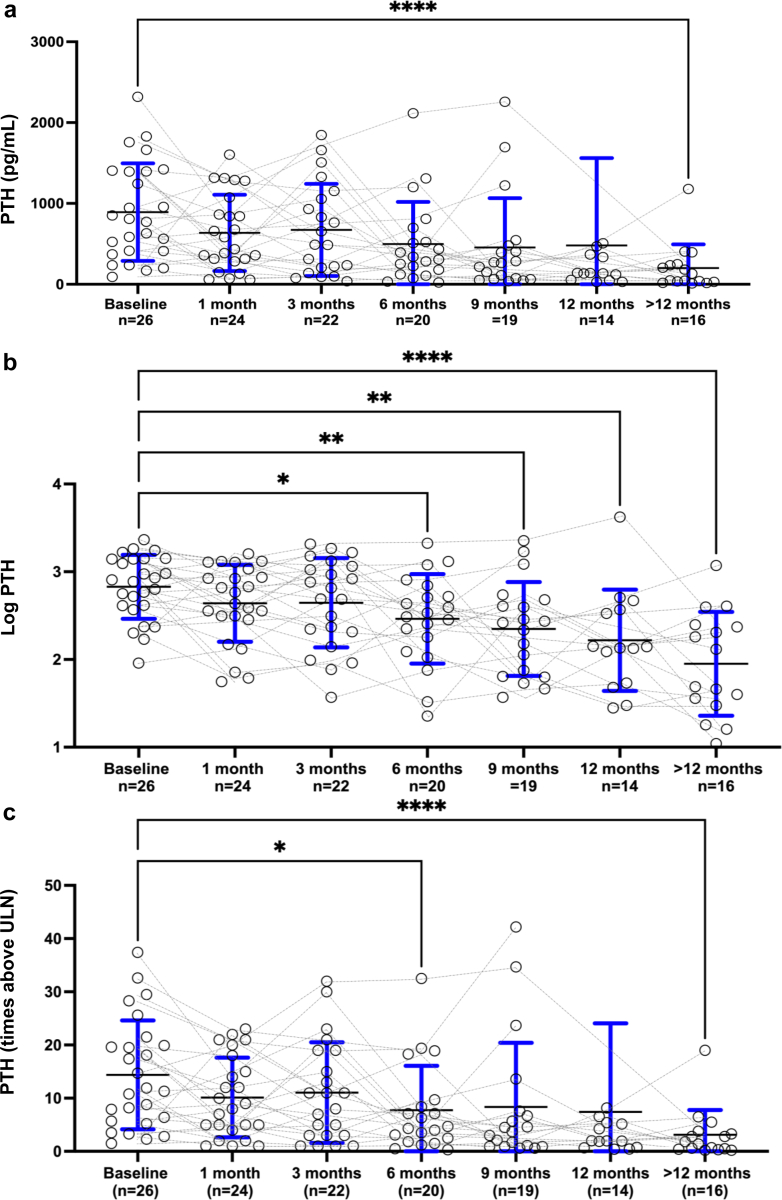
(a) Serum PTH, (b) log-PTH, and (c) PTH times above upper limit of normal at cinacalcet initiation and during follow-up. Individual values (circles) and median and quartiles are for each time-point. ∗*P* < 0.05, ∗∗*P* < 0.01, ∗∗∗*P* < 0.001, and ∗∗∗∗*P* < 0.0001 (linear mixed model (REML) followed by Dunnett’s multiple comparisons tests compared to baseline). PTH, parathyroid hormone.

**Figure 2:**
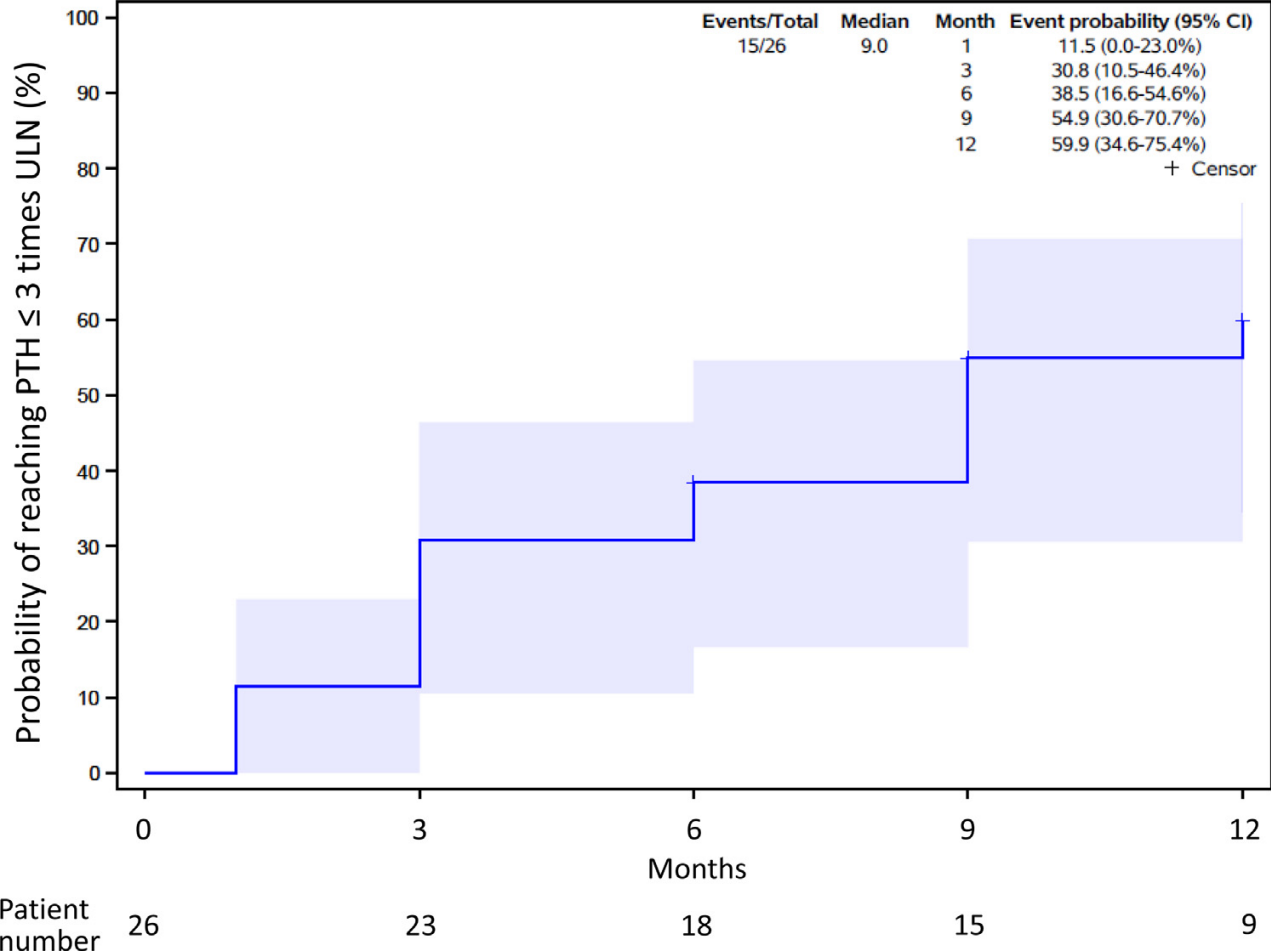
Probability of achieving a serum PTH below 3 times the upper limit of normal. Kaplan-Meier survival analysis demonstrating the time to achieve parathyroid hormone (PTH) levels below 3 times the upper limit of normal (ULN), that is, the target range according to European guidelines.^6^ The X-axis gives cinacalcet treatment times, the Y-axis the probability, the blue shaded area the 95% confidence interval.

**Figure 3:**
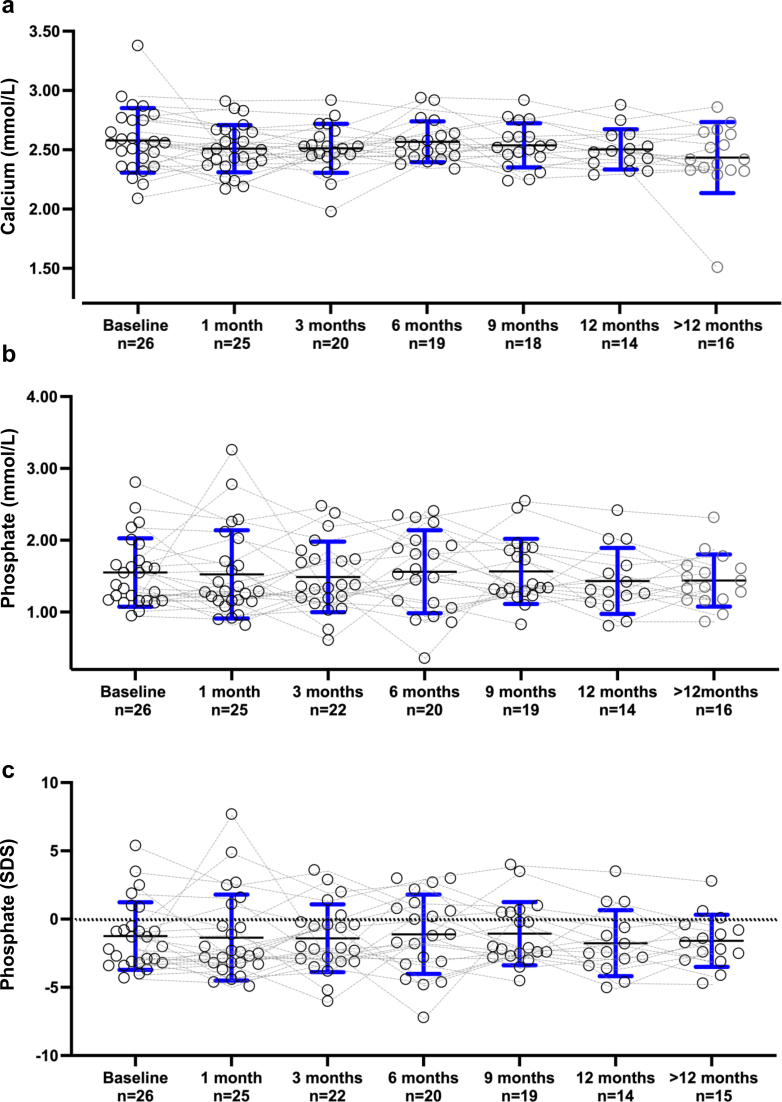

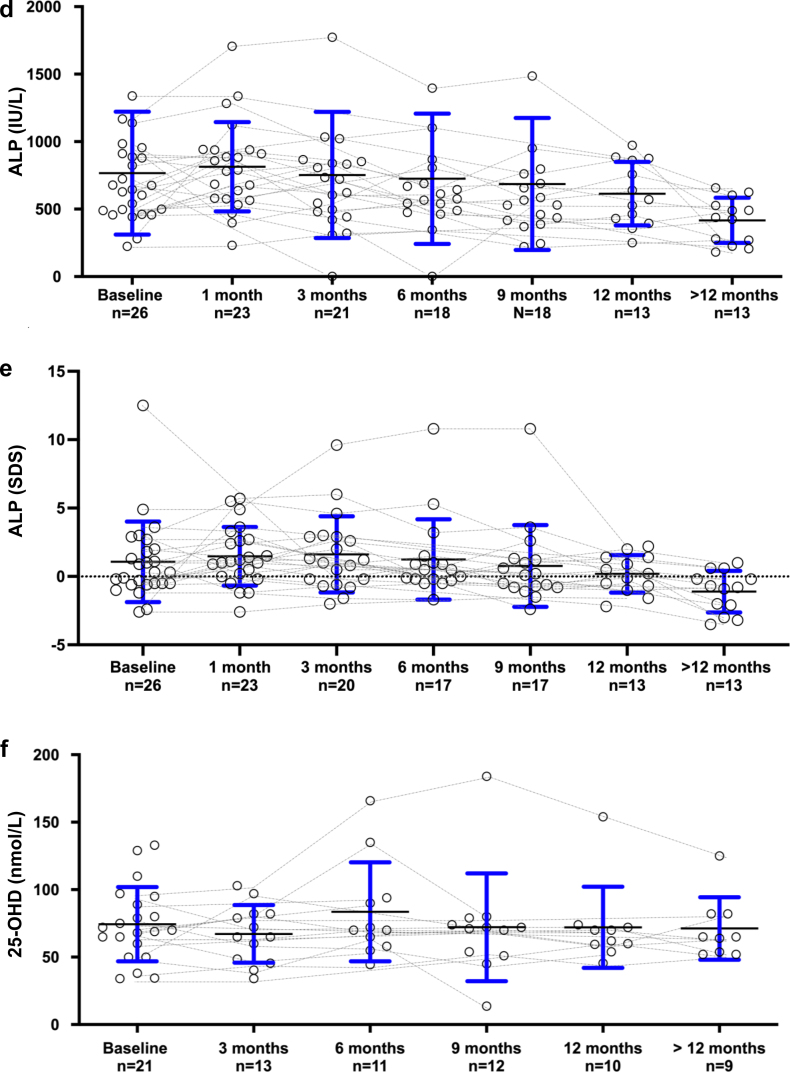
Comparison of (a) corrected calcium levels, (b) phosphate, (c) phosphate levels as SD score (SDS) for age, (d) ALP, (e) ALP levels as SDS for age, and (f) 25-OH vitamin D levels data at cinacalcet initiation and during follow-up. Individual values (circles) and median and quartiles are for each time-point. *P* = not statistically significant (NS) and ∗∗*P* < 0.01. (linear mixed model (REML) followed by Dunnett’s multiple comparisons tests compared to baseline).

Biochemical evolution is displayed in Table 2 and Figure 1, Figure 2, Figure 3. During cinacalcet treatment, PTH levels steadily declined to 4.3 (2.2–7.8), 2.0 (1.0–5.3) and 1.6 (0.5–3.4) times ULN after 6 months, 12 months, and last follow-up, respectively (*P* = 0.017, 0.003, and <0.0001, log-transformed PTH). The probability of achieving serum PTH in the target range (i.e., <3 times ULN) increased over time and was above 50% after 9 months. Subgroup analyses in patients receiving calcium-based binders as compared to patients receiving noncalcium-based binders did not show significant differences for the evolution of PTH levels.

**Table 2:** Cinacalcet dose, calcium and phosphate intake and biochemical findings at baseline and during follow-up

	Baseline *n* = 26	1 month *n* = 25	3 months *n* = 22	6 months *n* = 20	9 months *n* = 19	12 months *n* = 14	>12 months *n* = 16
Cinacalcet dose (mg/kg/d)	0.4 (0.2–0.8)	0.5 (0.3–1.0)	0.8 (0.3–1.1)	0.9 (0.5–1.1)	0.9 (0.4–1.0)	0.6 (0.4–0.9)	1.0 (0.4–1.2)
Oral Ca intake (% RNI)	84 (66–117)	100 (63–127)	100 (64–142)	93 (78–135)	85 (59–101)	78 (65–103)	76 (55–104)
P intake (% RNI)	47 (40–52)	48 (41–56)	40 (35–51)	48 (45–58)	48 (45–61)	47 (41–51)	62^a^ (57–85)
PTH (pg/ml)	792 (411–1397)	509 (307–916)	490 (207–1056)	323 (165–566)	262 (70–443)	138 (71–361)	90^d^ (35–239)
Log-PTH (pg/ml)	2.9 (2.6–3.1)	2.7 (2.5–3.0)	2.7 (2.3–3.1)	2.5^a^ (2.1–2.8)	2.4^b^ (1.8–2.7)	2.1^b^ (1.7–2.6)	1.9^d^ (1.5–2.4)
PTH (times ULN)	11.6 (5.9–19.8)	8.7 (4.7–15.8)	8.0 (3.3–14.7)	4.3^a^ (2.2–7.8)	3.7 (1.0–7.2)	2.0 (1.0–5.3)	1.6^d^ (0.5–3.4)
Log-PTH (times ULN)	1.1 (0.7–1.3)	0.9 (0.6–1.2)	1.0 (0.5–1.3)	0.7^a^ (0.3–1.0)	0.5^b^ (0.0–0.9)	0.3^b^ (−0.1 to 0.8)	0.2^c^ (−0.4 to 0.6)
Calcium (mmol/l)	2.56 (2.43–2.75)	2.47 (2.38–2.60)	2.51 (2.45–2.58)	2.54 (2.45–2.63)	2.52 (2.44–2.61)	2.48 (2.39–2.53)	2.42 (2.33–2.64)
P (mmol/l)	1.47 (1.16–1.71)	1.28 (1.16–1.62)	1.37 (1.21–1.83)	1.51 (1.08–1.98)	1.34 (1.26–1.90)	1.34 (1.13–1.74)	1.44 (1.23–1.63)
P (SDS)	−1.7 (−3.2 to −0.6)	−2.5 (−3.2 to −0.5)	−2.1 (−3.1 to −0.3)	−1.2 (−3.2 to 0.9)	−2.1 (−2.6 to 0.5)	−2.5 (−3.3 to −0.8)	−1.7 (−2.8 to −0.6)
25-D (nmol/l)	70 (60–89)	NA	65 (49–82)	70 (62–92)	72 (53–75)	66 (60–72)	65 (54–82)
ALP (IU/l)	660 (492–905)	789 (579–939)	728 (492–857)	613 (540–805)	548 (421–744)	570 (430–861)	438 (270–526)
ALP (SDS)	0.3 (−0.5 to 2.0)	1.1 (0.1–2.7)	1.1 (−0.3 to 2.9)	0.5 (−0.2 to 1.0)	−0.2 (−0.8 to 1.2)	0.2 (−0.7 to 1.4)	−0.8^b^ (−2.1 to −0.2)

25-D, 25-OH vitamin D; ALP, total alkaline phosphatase; Ca, calcium; NA, not available; P, phosphate; PTH, parathyroid hormone; RNI, reference nutrient intake; SDS, SD score; ULN, upper limit of normal.

Linear mixed model (REML) followed by Dunnett’s multiple comparisons tests compared to baseline were used for biochemical data.

a *P* < 0.05

b *P* < 0.01

c *P* < 0.001

d *P* < 0.0001

Albumin-corrected calcium levels remained within the normal range during the follow-up in the majority of patients. Serum phosphate (absolute and SDS) were low normal and remained stable. Total ALP and 25-OHD concentrations were mostly within the normal range. At last follow-up, ALP SDS was significantly lower compared to baseline (*P* = 0.004).

X-ray analyses were available for 23 patients: 13 patients presented with radiological signs of MBD including rickets, bone deformities and osteomalacia before cinacalcet initiation. Two patients suffered from low-trauma fractures of long bones during follow-up. One child suffered from a brown tumor of the femur.

### Safety of Cinacalcet and Growth in Infants

Infants on dialysis frequently suffer from nausea and vomiting, but no additional gastrointestinal symptoms were reported. During the cumulative observation period of 35.5 patient-years, 7 of the 26 children developed 10 hypocalcemic episodes (9 asymptomatic) after 8 (2–14) months and a cinacalcet dose of 1.5 (0.9–3.2) mg/kg/d (Table 3). Compliance with calcium supplements was not good in patient 22, leading to 1 hypocalcemic convulsion. Patient 19 developed 4 hypocalcemic episodes. At the time of hypocalcemic episodes, daily calcium intake was 104% (86%–127%) of RNI, PTH was 12.3 (5.4–19.6) ULN. Albumin-corrected calcium was 1.79 (1.76–1.97) mmol/l, phosphate SDS −2.7(−3.0 to −0.8). The dose of cinacalcet (mg/kg/d) was almost 2-fold higher at the time of hypocalcemia than the corresponding dose in the 19 children without hypocalcemia (Supplementary Table S1). Cinacalcet was discontinued in 3 children (patients 9, 16, 19) secondary to the hypocalcemic episodes after 1.7, 0.1, and 0.3 years under treatment (0.6, 0.2, and 3.5 mg/kg/d), respectively.

**Table 3:** Comparison of patients with and without hypocalcemic episodes

	Without hypocalcemic episodes (*n* = 12)	With hypocalcemic episodes (*n* = 7)	*P*
Patient characteristics at initiation			
Age (mo)	16 (11–21)	21 (13–23)	0.73
Height SDS	−1.6 (−2.3 to −0.7)	−1.7 (−2.4 to −1.3)	0.94
Body weight SDS	−1.2 (−2.2 to 0.1)	−1.3 (−2.2 to −0.2)	0.85
Total Ca supply (%RNI)	84 (68–96)	85 (51–119)	0.74
Cinacalcet dose	0.4 (0.2–0.8)	0.5 (0.3–1.0)	0.44
Maximum dose	1.0 (0.6–1.1)	1.2 (0.9–1.7)	0.31
Serum biochemistry at initiation			
Ca (mmol/l)	2.53 (2.36–2.64)	2.61 (2.51–2.73)	>0.99
Phosphate SDS	−1.1 (−3.2 to 0.8)	−2.2 (−3.5 to −1.7)	0.31
PTH (ng/l)	698 (414–1029)	631 (467–1530)	0.65
PTH (times ULN)	11.6 (6.2–17.6)	8.8 (6.5–22.5)	0.84
ALP SDS	1.1 (−0.3 to 3.2)	1.1 (0–1.9)	0.65
25-D (nmol/l)	68 (55–84)	70 (65–75)	0.89
Patient characteristics at hypocalcemic episodes			
Follow-up (mo)	9	8 (2–14)	
Age (mo)	25 (20–30)	32 (28–35)	0.34
Total calcium supply (%RNI)	85 (59–98)	104 (86–127)	0.16
Oral Ca supply	*n* = 5 (45%)	*n* = 4 (57%)	0.69
Cinacalcet dose	0.8 (0.4–0.9)	1.5 (0.9–3.2)	0.03
Active vitamin D dose	0.7 (0.4–1.2)	1.0 (0.6–1.1)	0.65
Serum biochemistry at hypocalcemic episodes			
Ca (mmol/l)	2.52 (2.41–2.58)	1.79 (1.76–1.97)	<0.0001
Phosphate SDS	−2.1 (−2.4 to 0.6)	−2.7 (−3.0 to −0.8)	0.31
PTH (ng/l)	340 (101–497)	884 (390–1407)	0.16
PTH (times ULN)	5.1 (1.5–9.1)	12.3 (5.4–19.6)	0.22
ALP SDS	−0.2 (−1.2 to 1.1)	1.4 (0.7–1.9)	0.08
25-D (nmol/l)	72 (54–74)	82 (70–82)	0.36

25-D, 25-OH vitamin D; ALP, total alkaline phosphatase; Ca, albumin corrected calcium; n, number; PTH, parathyroid hormone; RNI, reference nutrient intake; SDS, SD score; ULN, upper limit of normal.

Nonparametric Mann-Whitney test was used for group comparison.

Three girls, (patients 7, 17, and 26) developed precocious puberty, that is, thelarche, after 27, 13, and 6 months of cinacalcet therapy. Ultrasounds demonstrated numerous ovarian follicles in all; the uterus size and adrenal findings were normal for age as were serum follicle-stimulating hormone, luteinizing hormone, and estradiol concentrations. In 2 girls, cinacalcet was stopped at kidney transplantation, 4 and 21 months after diagnosis of precocious puberty. Symptoms persisted after transplantation in both, 1 was treated with gonadotropin-releasing hormone agonist. The third girl still had cinacalcet at the last follow-up, 8 months after diagnosis of precocious puberty, without progression of thelarche. Their biochemical evolutions are reported in Supplementary Table S2.

Body length SDS was largely stable with cinacalcet, and −1.7 (−2.5 to −0.8) at the start, −1.2 (−2.0 to −0.6) after 12 months, and −1.5 (−1.8 to −1.2) at last follow-up. Seven patients concomitantly received growth hormone therapy. Body weight SDS also remained stable and was −0.5 (−0.9 to −0.2) at baseline, −0.7 (−1.0 to −0.1) at 12 months, and −0.5 (−0.9 to −0.2) at last follow-up.

## Discussion

This comprehensive study on the off-label use of cinacalcet in pediatric dialysis patients aged <3 years demonstrates an efficient control of sHPT but also a risk of hypocalcemia in these rapidly growing patients, and a potential risk of precocious puberty. At the beginning of cinacalcet, serum PTH levels were 12-fold above the ULN. PTH levels steadily declined with cinacalcet, with more than 50% of patients reaching PTH levels in the target range after 9 months. The slow but efficient control of sHPT is similar to the one observed in older children on dialysis,^26^ and in children with primary hyperparathyroidism.^40^ In patients receiving cinacalcet for 12 months, PTH was reduced by about 80%. The cinacalcet starting dose was 2-times higher than the initial doses administered in the industry-sponsored trials and the starting dose of cinacalcet of 0.2 mg/kg/d recommended by the 2020 guidelines. However, cinacalcet was started between 2009 and 2021, mostly before guidelines publication.^43^ These doses are not justified by pharmacokinetic data.^41^, ^42^, ^43^ Conversely, the maximal doses used was in the range of the trials and the guidelines,^42^ except for 1 patient.

Hypocalcemia was observed in 27% of patients, of which 6 children had single asymptomatic episodes, whereas 1 child not adhering to the prescribed medication experienced symptomatic hypocalcemia. In the pediatric phase 2 and 3 randomized controlled trial, hypocalcemia was observed in 23% of patients in the cinacalcet group versus 19% in the placebo group.^26^ A fatal event occurred during the double-blind period in a teenage girl with prolonged QT interval, hypocalcemia, and medication interactions. Overall, the hypocalcemia incidence relative to treatment time in infants was not higher than in older children.

RNI of calcium should be at least 100% in children with CKD 2 to 5 of RNI in healthy children, with an upper safety threshold of 200%.^4^^,^^6^ Recent observational studies, however, yielded lower dietary calcium intake in the majority of children with CKD 3 to 5D and an inverse correlation with serum PTH.^10^^,^^11^ However, when considering calcium from medications, 41% of the children exceeded 200% of the recommended intake,^11^ whereas in our cohort 14 children received less than 100% of daily RNI for calcium at cinacalcet introduction. In clinical routine, calcium supply is difficult to assess and provides rough estimates only. Twenty percent to 40% of total formula calcium is absorbed, depending on the native and active vitamin D supply; however, it may be much lower with high oral calcium intake because intestinal calcium absorption follows a saturation curve.^44^ Extrapolating the total calcium intake of the infants to an adult of 75 kg body weight, baseline median calcium intake from diet and medication was 3255 (2477–4691) mg/d, that is, higher than in most adult dialysis patients, and the active vitamin dose was 14.5 (8.7–20.7) μg thrice weekly, which is close to the highest doses considered in calcium uptake modelling in adults.^44^ Fitting our findings in the infants to the calculations in adults, and ignoring differences in diets, intestinal uptake rate in the young children should be about 10% only, that is, about 325 mg/d. These extrapolations lack scientific data for patients with CKD and may introduce significant imprecision. At least in healthy infants, intestinal calcium uptake follows similar saturation curves as in adults.^45^ The relative contribution of dialytic calcium uptake in the children on peritoneal dialysis studied here and extrapolated to the adult model was 181 (127–139) mg/d, that is, dialytic calcium uptake increased net total calcium gain by about 55%. Thus, the relative low average oral calcium intake relative to the 100% to 200% RNI recommended^4^ may have been compensated by a relatively high dialytic calcium uptake. In line with this, 8 of the 26 children (31%) had serum calcium levels above the ULN for age at baseline, and 2 of the 14 children (14%) still after 1 year of cinacalcet therapy. Hypercalcemia carries a significant risk of adynamic bone disease and vascular calcification and requires stepwise reduction in calcium supply and vitamin D analogs,^4^ even though in infants, it is rather recommended to keep calcium within the normal or high normal range.^6^ Of note, 77 % of children exhibited hypophosphatemia at the time of cinacalcet initiation and remained stable (79%) during the 1-year follow-up, which is an additional risk factor for mineralization defects;^46^ the presence of hypophosphatemia in such populations is quite a novel finding, and may indicate either an overtreatment with phosphate binders, either a too restricted diet (subsequently also restricting calcium intake) or both. These findings demonstrate the challenges and the critical importance of the mineral balance in infants on dialysis: phosphate levels should be in the normal range for age (not too high but not too low). No systematic studies have been performed, which leaves the pediatric nephrologists with an individual approach, closely monitoring serum calcium, phosphate, and PTH; and considering trends over time. Cinacalcet may be started in children aged <3 years in case of persistent sHPT despite optimized conventional management and in the presence of high or high-normal calcium levels, that is, in patients with the most severe form of CKD-MBD, as suggested by the recent European guidelines,^6^ provided parents’ informed consent has been obtained and there is adequate therapy adherence to the cinacalcet and sometimes calcium supplementation. In view of the more than 80% reduction in serum PTH and the associated major influx of calcium into bone, increasing oral and dialytic calcium supply should regularly be reconsidered, especially in cases of declining serum calcium.

Different from previous small studies, height SDS did not significantly improve, despite major reduction of exceedingly high PTH values.^27^^,^^32^ This suggests that control of PTH has no major effect on growth, which is in line with observations from the International Pediatric Peritoneal dialysis Network registry with large patient numbers.^37^ Although our data did not show a significant impact of cinacalcet on longitudinal growth, this conclusion must be considered speculative due to the missing data, the short follow-up time and the use of recombinant growth hormone in 7 patients.

Three of the young children on cinacalcet, however, developed precocious puberty, though pubertal development is usually delayed in children on dialysis.^47^ They had congenital abnormalities of the kidney and urinary tract. Two of them underwent genetic testing, with pathogenic HNF1β variants. In 2009, precocious puberty was reported in a 5-year old-boy with CKD5 due to HNF1β variants who was on cinacalcet.^48^ The mechanistic link between precocious puberty and cinacalcet therapy is unknown; however, these findings indicate the need for close monitoring for endocrine changes in children receiving cinacalcet, especially in presence of HNF1β variants.

This largest cohort study of cinacalcet use in infants undergoing maintenance dialysis has strengths; notably, quite detailed data on nutrition, medications, doses, and clinical evolution but also has some limitations, due to its retrospective design. The assessment of nutrition calcium was not standardized among centers, and, in the absence of respective pediatric data, calculation of the intestinal calcium uptake had to be based on data established in adults; dialytic calcium uptake was estimated based on average values obtained in a randomized controlled trial in children.^34^ This provides rough estimates of the true calcium balance only and highlights the need of further research. Ionized calcium was not widely available, despite the fact that the combination of antacid drugs, vomiting, and alkalinization often seen in infants may modify the free-to-ionized ratio, and further decrease ionized calcium.^6^

In conclusion, our findings provide evidence for efficient control of severe sHPT in children aged <3 years by cinacalcet. It requires close monitoring of calcium and phosphate balance and adaptation of the increasing calcium requirements in these rapidly growing children with the most severe forms of CKD-MBD. This treatment should be initiated and managed by experienced pediatric nephrologists. The link between precocious puberty and cinacalcet is intriguing and deserves a close endocrine follow-up.

## Disclosure

JBa received consulting fees from Amgen and Bayer, and research fees from Amgen; and is an investigator of the cinacalcet and etelcalcetide Amgen-sponsored trials. DH received speaker and/or consulting fees from Biologix, Chiesi, and Kyowa Kirin; and research grants from Amgen, Chiesi, and Kyowa Kirin. CPS received consultancy honoraria from Baxter, Iperboreal, and STADAPHARM, and lecturing honoraria from Fresenius; and is investigator of the Amgen-sponsored cinacalcet and etelcalcetide trials. RS received research grants from Fresenius Medical Care and consultancy fees and/or speaker honoraria from Amgen, Fresenius Medical Care, Astra Zeneca, Vitaflo, and Humacyte.

IZ received lecturing honoraria from Baxter and Vitaflo. All the other authors declared no competing interests.

## Supplementary Material

Supplementary File (PDF) **Table S1.** Patient age, cinacalcet treatment and biochemical findings of children during hypocalcemic episodes (calcium < 2.10 mmol/l). **Table S2.** Cinacalcet dose, calcium intake and biochemical findings at baseline and during follow-up for patients with HNF1B pathogenic mutations. **Checklist.**

## Footnotes

Supplementary File (PDF)
**Table S1.** Patient age, cinacalcet treatment and biochemical findings of children during hypocalcemic episodes (calcium < 2.10 mmol/l) **Table S2.** Cinacalcet dose, calcium intake and biochemical findings at baseline and during follow-up for patients with HNF1B pathogenic mutations
**Checklist.**
