# Supplementary material for: Safety and Efficacy of Cinacalcet in Children Aged Under 3 Years on Maintenance Dialysis
Source: Kidney Int Rep. 2024 May 7;9(7):2096–109. doi: 10.1016/j.ekir.2024.04.061 (PMC11284406; doi:10.1016/j.ekir.2024.04.061)
Supplement: Supplementary File (PDF) — Table S1. Patient age, cinacalcet treatment and biochemical findings of children during hypocalcemic episodes (calcium < 2.10 mmol/l). Table S2. Cinacalcet dose, calcium intake and biochemical findings at baseline and during follow-up for patients with HNF1B pathogenic mutations. Checklist. [file mmc1.pdf]

Supplemental Tables

Supplemental table S1: Patient age, cinacalcet treatment and biochemical findings of children during hypocalcemic episodes (Ca < 2.10 mmol/L)

**Pat. Nº**	**Patient Age** (months)	**Cina treatment** (years)	**Cina dose** (mg/kg/d)	**Body weight** (kg)	**Total Ca intake** (mg/d)	**Ca_c** (% RNI)	**P intake** (mg/d)	**Ca based binders**	**Non Ca based binders**	**Alfacalcidol** (µg/day)	**Dialysate Ca** (mmol/L)	**Corrected serum Ca** (mmol/L)	**Serum P** (mmol/L)	**P** (SDS)	**PTH** (pg/mL)	**PTH** (times > ULN)
**3**	11	0.2	0.6	9.4	603	128	220	Ca suppl	No	1	1.75	1.99	1.76	-0.3	769	10.7
**4**	27	1.3	0.8	11.6	1059	183	424	Ca suppl	No	1	1.75	1.76	1.38	-2.0	852	11.9
**9**	33	1.9	1.2	12.4	691	111	375	No	Sevelamer	0.2	1.25	1.51	1.18	-3.0	36	0.5
**16**	21	0.1	0.2	6.2	3525	671	NA	Ca suppl	Sevelamer	1.2	1.75	1.70	1.73	-0.4	2420	37.2
**17**	36	3.2	2.3	12.8	NA	NA	NA	No	Sevelamer	1.5	1.25	2.02	1.13	-3.3	5511	88.9
**19**	29	0.3	1.6	9.2	483	105	197	Yes	Sevelamer	0.8	1.75	1.97	1.28	-2.5	1103	15.4
**19**	33	0.7	3.7	10.8	489	91	197	No	No	0	1.75	1.79	1.01	-3.9	198	3.7
**19**	35	0.8	3.7	10.8	491	91	241	No	No	0.8	1.75	1.95	1.99	1.1	916	12.8
**19**	37	1.0	3.5	11.5	490	85	241	No	No	0.2	1.75	1.78	1.21	-2.9	263	3.7
**22**	30	0.2	1.3	11.4	842	148	285	Yes	No	1	1.75	1.98	1.2	-2.9	1508	21.1

Abbreviations: Nº, number; Cina, Cinacalcet; Ca_c, albumin corrected calcium; RNI, Reference Nutrient Intake; P, phosphate; Suppl, supplementation; ULN, upper limit of normal; SDS, Standard Derivation Score; NA, Not available. Total Ca includes Ca intake with diet and medication and dialytic Ca gain

Supplemental Table S2: Cinacalcet dose, calcium intake and biochemical findings at baseline and during follow-up for patients with HNF1B pathogenic mutations

	**Baseline**			**1 month**			**3 months**			**6 months**			**9 months**			**12 months**			**Last FU**		
**Patients**	**7**	**17**	**26**	**7**	**17**	**26**	**7**	**17**	**26**	**7**	**17**	**26**	**7**	**17**	**26**	**7**	**17**	**26**	**7 (2.6yo)**	**17 (3.6yo)**	**26 (1.2yo)**
**Cinacalcet dose** (mg/kg/d)	0.1	0.3	0.2	0.6	0.5	0.3	0.6	0.5	0.3	0.9	0.5	0.5	0.8	0.4	1.0	1.2	0.4	0.9	0.4	2.1	1.1
**Oral Ca intake** (% RNI**)**	968	44	66	650	53	NA	650	53	NA	837	52	30	768	47	NA	396	59	21	286	76	21
**PTH** (pg/mL)	957	2320	1757	NA	1847	655	NA	1847	655	2115	522	393	2258	54	286	4196	507	337	249	1178	405
**PTH** **(**times ULN)	14.7	37.4	28.3	NA	29.8	10.6	NA	29.8	10.6	32.5	8.4	6.3	34.7	0.9	4.6	64.6	8.2	5.4	3.8	19.0	6.5
**Calcium** (mmol/L)	2.55	2.75	2.80	2.45	2.47	2.66	2.45	2.47	2.66	2.51	2.64	2.94	2.52	2.78	2.92	2.32	2.53	2.75	2.52	2.29	2.86
**P** (mmol/L)	1.72	1.16	2.45	2.20	1.38	2.00	2.20	1.38	2.00	2.35	1.16	2.32	1.21	1.29	2.55	2.02	1.54	2.42	1.28	1.16	2.32
**P** (SDS)	-0.5	-3.7	3.5	2.0	-2.0	1.4	2.0	-2.0	1.4	2.7	-3.1	3.0	-3.0	-2.2	4.0	1.3	-1.2	3.5	-2.5	-3.1	2.8
**25-D** (nmol/L)	34	75	38	34	NA	46	34	NA	45	NA	NA	45	51	NA	54	62	NA	46	52	65	52

Abbreviations: SDS, standard deviation score; Ca, calcium; RNI, Reference Nutrient Intake; P, phosphate ULN, upper limit of normal; 25-D, 25-OH vitamin D; e; NA, not available.
